# Chitosan nanoparticles loaded with the antimicrobial peptide temporin B exert a long-term antibacterial activity *in vitro* against clinical isolates of *Staphylococcus epidermidis*

**DOI:** 10.3389/fmicb.2015.00372

**Published:** 2015-04-28

**Authors:** Anna M. Piras, Giuseppantonio Maisetta, Stefania Sandreschi, Matteo Gazzarri, Cristina Bartoli, Lucia Grassi, Semih Esin, Federica Chiellini, Giovanna Batoni

**Affiliations:** ^1^Department of Chemistry and Industrial Chemistry, University of PisaPisa, Italy; ^2^National Interuniversity Consortium of Materials Science and Technology, FlorenceItaly; ^3^Department of Translational Research and New Technologies in Medicine and Surgery, University of PisaPisa, Italy

**Keywords:** antimicrobial peptides, temporin B, delivery system, chitosan, nanoparticles, *Staphylococcus epidermidis*

## Abstract

Nowadays, the alarming rise in multidrug-resistant microorganisms urgently demands for suitable alternatives to current antibiotics. In this regard, antimicrobial peptides (AMPs) have received growing interest due to their broad spectrum of activities, potent antimicrobial properties, unique mechanisms of action, and low tendency to induce resistance. However, their pharmaceutical development is hampered by potential toxicity, relatively low stability and manufacturing costs. In the present study, we tested the hypothesis that the encapsulation of the frog-skin derived AMP temporin B (TB) into chitosan nanoparticles (CS-NPs) could increase peptide’s antibacterial activity, while reducing its toxic potential. TB-loaded CS-NPs with good dimensional features were prepared, based on the ionotropic gelation between CS and sodium tripolyphosphate. The encapsulation efficiency of TB in the formulation was up to 75%. Release kinetic studies highlighted a linear release of the peptide from the nanocarrier, in the adopted experimental conditions. Interestingly, the encapsulation of TB in CS-NPs demonstrated to reduce significantly the peptide’s cytotoxicity against mammalian cells. Additionally, the nanocarrier evidenced a sustained antibacterial action against various strains of *Staphylococcus epidermidis* for at least 4 days, with up to 4-log reduction in the number of viable bacteria compared to plain CS-NPs at the end of the observational period. Of note, the antimicrobial evaluation tests demonstrated that while the intrinsic antimicrobial activity of CS ensured a “burst” effect, the gradual release of TB further reduced the viable bacterial count, preventing the regrowth of the residual cells and ensuring a long-lasting antibacterial effect. The developed nanocarrier is eligible for the administration of several AMPs of therapeutic interest with physical–chemical characteristics analog to those of TB.

## Introduction

Over the last years, nanotechnologies have undergone a rapid development in many different fields, including microbiology and infectious diseases (Seil and Webster, 2012). In particular, nanostructures hold enormous potential as effective drug-delivery systems to treat microbial infections as they exhibit several advantages as compared to conventional antibiotic formulations (Gao et al., 2011). For instance, NPs may be endowed of an inherent antimicrobial activity that may sum/synergize to that of the loaded antibiotic; furthermore, they often display high degree of biocompatibility, possibility to incorporate both lipophilic and hydrophilic drugs, ability to release the loaded antimicrobial with controlled rate and continued kinetics protecting it from degradation, while reducing its side effects.

Nowadays, microbial resistance to antibacterial and antifungal drugs has reached critical levels, invalidating the therapeutic efficacy of a large proportion of molecules currently used in the clinic (Alanis, 2005; Fair and Tor, 2014). In this regard, AMPs have been repetitively proposed as a valid alternative to conventional antibiotics (Lohner, 2009; Batoni et al., 2011; Yeung et al., 2011; Aoki and Ueda, 2013; Roscia et al., 2013). They comprise a large and diverse group of molecules with low tendency to induce bacterial resistance and endowed with strong and fast antimicrobial activity, directed also toward multi-drug-resistant microorganisms (Zasloff, 2002; Maisetta et al., 2006; Del Gaudio et al., 2013). Nevertheless, despite the initial enthusiasm for the possible use of AMPs as new class of antibiotics, their clinical use remains limited because of their poor stability in biological fluids, undesirable interactions with host macromolecules, and potential toxicity (Brogden and Brogden, 2011; Kang et al., 2014). Because of these limitations, it is becoming progressively clear that the use of natural peptides as such is very unlikely to result in therapeutic applications and that peptide modifications and/or development of appropriate peptide formulations are needed to improve the stability, delivery, and efficacy of promising molecules. In this regard, the encapsulation of AMPs in micro–nanostructures might represent an innovative approach to overcome the problems that still limit AMP-clinical use, allowing stability of the encapsulated peptide, its safeguard from proteolytic degradation, control of the pharmacokinetics, and reduced toxicity. Such strategies are currently an emerging and promising field of investigation (Yadav et al., 2011; Brandelli, 2012).

Chitosan is a high-molecular-weight linear polycationic heteropolysaccharide comprising copolymers of β-1,4-linked D-glucosamine and *N*-acetyl-D-glucosamine obtained by deacetylation of its parent polymer chitin, the second most abundant natural polymer in nature after cellulose, found in the structure of a wide number of invertebrates and in the cell walls of fungi (Duttagupta et al., 2015). CS has proved to be biocompatible, non-toxic, biodegradable, and a safe excipient in drug formulation over the last decades (Thanou et al., 2001; Duttagupta et al., 2015). It also possesses mucoadhesive properties and exerts antimicrobial activity against a broad spectrum of microorganisms including Gram-positive and Gram-negative bacteria, filamentous fungi and yeast (Kong et al., 2010). All these characteristics render CS a promising candidate for encapsulation of antimicrobial compounds and for the development of novel nano-therapeutics to treat microbial infections.

We have previously explored with success the possibility to use CS-NPs as delivery systems of proteins with antibacterial activity such as lysozyme (Piras et al., 2014). The suitability of CS-NPs for the controlled release of AMPs was further investigated by us selecting RSI as a fluorogenic peptide model for cationic amphiphilic peptides (Piras et al., 2015). These studies demonstrated that the CS-NPs formulation, developed in presence of the model cationic peptide, had “core-shell” features allowing for the linear release of the encapsulated peptide, an encapsulation efficacy up to 98%, and possessed the high cytocompatibility typical of CS based nanocarriers. Based on these encouraging results, the present study was focused on the application of the developed model nanocarrier, for the loading and release of the cationic peptide TB, which share with RSI key chemical–physical properties that have great influence in the NPs formulation process. These include the same positive net charge at neutral pH (+2), similar percent of hydrophobic amino acids (62.5 vs. 61%) and comparable dimensions (1301 vs. 1393 Da). TB belongs to a large family of small, alpha-helical, and mildly cationic AMPs isolated from amphibian skin secretions and endowed with broad spectrum antimicrobial activity (antibacterial, antiviral, antifungal and, in some cases, anti-parasitic), high cytocompatibility, and immune-modulating properties (Mangoni et al., 2005; Mangoni, 2006). TB has previously shown a strong and fast killing ability, especially directed against Gram-positive, multi-drug-resistant nosocomial bacterial species (Mangoni et al., 2008).

The antibacterial properties of the developed TB-CS-NPs were tested against *Staphylococcus epidermidis*, a bacterial species chosen as model Gram-positive bacterium, increasingly involved in opportunistic infections in hospital settings (Gomes et al., 2014). Appropriate experimental conditions were set up in order to evaluate long-term killing ability of TB-CS-NPs. We found that, beyond the intrinsic antibacterial activity of either CS-NPs or TB alone, the loaded nanocarrier showed a potent, and prolonged bactericidal ability against various *S. epidermidis* strains. In addition, investigation of the cytocompatibility of free TB vs. that of the peptide loaded into CS-NPs, highlighted an excellent role of the nanocarrier in reducing the peptide’s toxicity toward mammalian cells, thus representing a promising model for the development of NPs-based delivery system of AMPs with therapeutic potential.

## Materials and Methods

### Materials

Chitosan (Medium molecular weight, Mw 108 kDa (Mw/Mn 2.4), Deacetylation Degree ∼92% (Piras et al., 2014) and sodium TPP were purchased from Sigma–Aldrich, Milan, Italy.

Synthetic TB (LLPIVGNLLKSLL-amide) was obtained from Peptide Specialty laboratories GmBH, Heidelberg, Germany.

Acetic acid analytical grade was purchased from Carlo Erba, Milan, Italy. Deionized water (Milli-Q, ddH_2_O) was used throughout the experiments. Micro BCA^TM^ Assay Kit was purchased from Thermo Scientific, Rockford, IL, USA. Mouse embryo fibroblast BALB/3T3 clone A31 (CCL-163) cell line was purchased from ATCC, (LGC standards, Milan, Italy) and propagated as indicated by the supplier. DMEM, 0.01 M pH7.4 Dulbecco’s phosphate buffer saline without Ca^2+^ and Mg^2+^, bovine calf serum, L-glutamine, and antibiotics (penicillin/streptomycin) were purchased from Gibco, (Monza, Italy). Cell proliferation reagent WST-1 was purchased from Roche Diagnostics (Milan, Italy).

### Preparation of Blank CS-NPs or TB-CS-NPs

Chitosan nanoparticles were prepared using a simple ionic gelation process as described earlier (Piras et al., 2015). Briefly, CS was dissolved in 1% (v/v) acetic acid (1 mg/ml, pH 5) and TPP was dissolved in water (1 mg/ml); for TB-CS-NPs preparation, 200 μg of the peptide were added to the CS solution. NPs formed spontaneously upon addition of 2 ml of TPP aqueous solution to 5 ml of the CS solution under magnetic stirring; the mixture was stirred at room temperature for 2 h.

Nanoparticles suspensions were purified by centrifugation in ALC^®^(Milan, Italy) PK121R centrifuge at 14000 *g* for 30 min or at 9000 *g* for 60 min, at 4°C.

### Characterization of NPs

The size distribution of the developed NPs was measured by means of dynamic light scattering (Coulter LS230 Laser Diffraction Particle Size Analyzer, Beckman Coulter, Nyon, Switzerland). The Zeta-potential of the developed formulations was evaluated using a Beckman-Coulter Delsa^TM^ Nano C, at 25°C in 10 mM SPB, pH 7.4.

### Evaluation of TB Loading Capacity and *In Vitro* Release Kinetics of NPs

The TB content present in the supernatants obtained from TB-CS-NPs purification was measured at 565 nm by means of Micro BCA^TM^ protein assay. The Loading content (L) was defined as the amount of TB per TB-CS-NPs dry weight; the encapsulation efficiency was defined as the amount of TB recovered in TB-CS-NPs compared to the total amount of peptide used in the formulation protocol.

Purified TB-loaded CS-NPs were re-dispersed in 1 ml of SPB pH 7.4 (300 μg/ml) and placed into test tubes at 37°C under magnetic stirring. At appropriate intervals, samples were centrifuged at 14000 *g* 4°C for 30 min, the supernatants were collected and replaced by 1 ml of fresh medium. The amount of TB released from the CS-NPs was evaluated by means of Micro BCA^TM^ protein assay. All CS-NP formulations and characterization testing were repeated at least three times.

### Biological Evaluations

#### Cytotoxicity Tests

Cytotoxicity evaluation of TB and TB-CS-NPs was carried out using BALB/3T3 clone A31 mouse embryo fibroblasts cell line. Cells were grown in complete DMEM containing 10% (v/v) calf serum, 4 mM L-glutamine, 100 U/ml of penicillin and 100 μg/ml of streptomycin. A subconfluent monolayer of BALB/3T3 fibroblasts was washed with Dulbecco’s phosphate buffered saline, trypsinized using a 0.25% trypsin, 1 mM EDTA solution, centrifuged at 200 *g* for 5 min, re-suspended in growth medium and counted. Appropriate dilution was made in order to obtain 3 × 10^3^ cells *per* 100 μl of medium, the final volume present in each well of a 96 well plate. Cells were incubated at 37°C, 5% CO_2_ for 24 h until 60–70% confluence was reached. For the determination of TB IC_50_ (50% inhibitory concentration, defined as the peptide concentration at which 50% of cell death in respect to the control is observed), cells were exposed to different concentrations of TB (3.64–466 μg/ml) dissolved in complete DMEM. Cytotoxicity assessment of the developed nanocarriers was performed by incubating BALB/3T3 cell line with medium containing TB-CS-NPs (2.5, 5, and 10 mg/ml, loaded with 116.5, 233, and 466 μg/ml of TB, respectively). Cells incubated with fresh complete DMEM were used as control and growth medium as blank. After 24 h of incubation, cells were analyzed for viability with Cell Proliferation Reagent WST-1, a yellow tetrazolium salt that is reduced to formazan only by metabolically active cells. Briefly, cells were incubated with WST-1 reagent diluted 1:10 for 4 h at 37°C, 5% CO_2_. Measurements of formazan dye absorbance were carried out at 450 nm, with the reference wavelength at 655 nm, by using a Bio-Rad Benchmark Microplate Reader (Bio-Rad, Hercules, CA, USA).

#### Bactericidal Assays

The *S. epidermidis* strains used in the study included a reference strain purchased from the ATCC (*S. epidermidis* ATCC 35984) as well as four clinical isolates obtained from blood cultures or central venous catheters at the Microbiology Unit of the University Hospital of Pisa, Italy (Brancatisano et al., 2014). The susceptibility profile of the different strains to conventional drugs is shown in **Table 1**. For preparation of stock cultures, the bacterial strains were grown in TSB (Oxoid Basingstoke, UK) at 37°C until mid-log phase, subdivided in aliquots, and kept frozen at -80°C until future use. For CFU count, serially diluted bacterial suspensions were plated on TSA (Oxoid, Basingstoke, UK) and incubated for 48 h at 37°C.

**Table 1:** Antibiotic susceptibility profiles of the different *Staphylococcus epidermidis* strains used in the study.

	ATCC 35984	Se30	SeBER	SeNOC	SeVIC
Ciprofloxacin	NT	≥8^a^	NT^b^	NT	NT
Clindamycin	≥4	≥8	0.5	≤0.25	≤0.25
Daptomycin	0.5	NT	NT	NT	NT
Erythromycin	≥8	≥8	≥8	≥8	≤0.25
Fosfomycin	NT	≤8	≤8	≤8	≤8
Fusidic Acid	≤0.5	≥32	≤0.5	≤0.5	≤0.5
Gentamicin	8	≥16	≤0.5	≤0.5	8
Levofloxacin	≤0.12	≥8	≥8	4	≤0.12
Linezolid	2	NT	2	1	1
Norfloxacin	NT	≥16	NT	NT	NT
Nitrofurantoin	NT	≤16	≤16	≤16	≤16
Oxacillin	≥4	≥4	≥4	≥4	≥4
Penicillin G	NT	≥0.5	≥0.5	≥0.5	≥5
Rifampicin	≤0.03	≤0.5	≤0.5	≤0.5	≤0.5
Teicoplanin	4	NT	4	4	4
Tetracycline	≤1	2	2	≥16	≤1
Tigecycline	≤0.12	NT	≤0.12	0.5	≤0.12
Tobramycin	NT	≥16	≥16	≤1	2
Trimethoprim/ Sulfamethoxazole	80	≥320	≤10	≤10	≤10
Vancomycin	2	NT	2	1	2

*^a^The numbers are the MIC values expressed in μg/ml; ^b^NT, Not Tested*.

The antibacterial activity of the developed TB-CS-NPs was compared to that exerted by the plain nanocarrier (CS-NPs) and by free TB. *S. epidermidis* cells, growing exponentially in TSB, were washed and re-suspended in 1 ml of SPB 10 mM, pH 7.4 at approximately 1 × 10^8^ CFU/ml. Bactericidal assays were performed in the same buffer, added with 1.25% TSB (v/v; buffer assay). The test samples were prepared by adding 10 μl of the bacterial suspension (1 × 10^6^ CFU) to 90 μl of the buffer assay containing TB-CS-NPs, CS-NPs, or free TB at optimal concentrations determined in preliminary experiments (data not shown). Samples containing only bacteria in the buffer assay were set-up as controls for cell-viability. All the samples were incubated at 37°C under agitation for a total of 4 days. After 4 h incubation and at 24 h intervals, aliquots from the samples were taken, serially diluted and plated on TSA for CFU counting. Results were expressed as log_10_ CFU/ml.

### Statistical Analysis

All the characterizations were performed at least on three replicates, unless otherwise specified. The data were statistically analyzed using Student’s *t*-test. Statistical significance was set at the level of ^∗^*p* < 0.05.

## Results

### Preparation and Characterization of TB-CS-NPs

Chitosan nanoparticles were easily obtained by means of ionic gelation between cationic CS and anionic TPP, as previously described (Piras et al., 2015). The formulation parameters (CS solution pH and concentration, amount of peptide) previously set for the model peptide RSI were applied to the encapsulation of TB in CS-NPs. The prepared NP suspensions appeared opalescent and without macroscopically appreciable aggregates. The dynamic light scattering analysis of the prepared NPs revealed that the loading of TB led to an increase in the NPs average diameter, being the obtained diameters 124 ± 17 and 185 ± 10 nm, for blank and TB-CS-NPs, respectively (**Table 2**).

**Table 2:** Main characteristics of plain CS-NPs and TB-CS-NPs: average diameter distribution (Size), yield of the formulations and purification processes (yield), surface charge (Zeta potential), loading capacity (L), and encapsulation efficiency (EE).

Formulation	Size (nm ± SD)	PdI^a^	Yield (% ± SD)	Zeta potential (mV ± SD)	L (% ± SD)	EE (% ± SD)
CS-NPs	124 ± 17	0.010	41.4 ± 0.2	+21.9 ± 0.6	–	–
TB-CS-NPs	185 ± 10	0.063	43.1 ± 0.1	+8.8 ± 0.1	4.8 ± 0.2	74.7 ± 2.3

^a^Polydispersity index of the diameter distribution peak.

Both blank and loaded NPs displayed positive Zeta potential values in SPB at pH 7.4, due to the cationic nature of CS. The TB loading led to a statistically significant decrease of the Zeta potential value.

**FIGURE 1:**
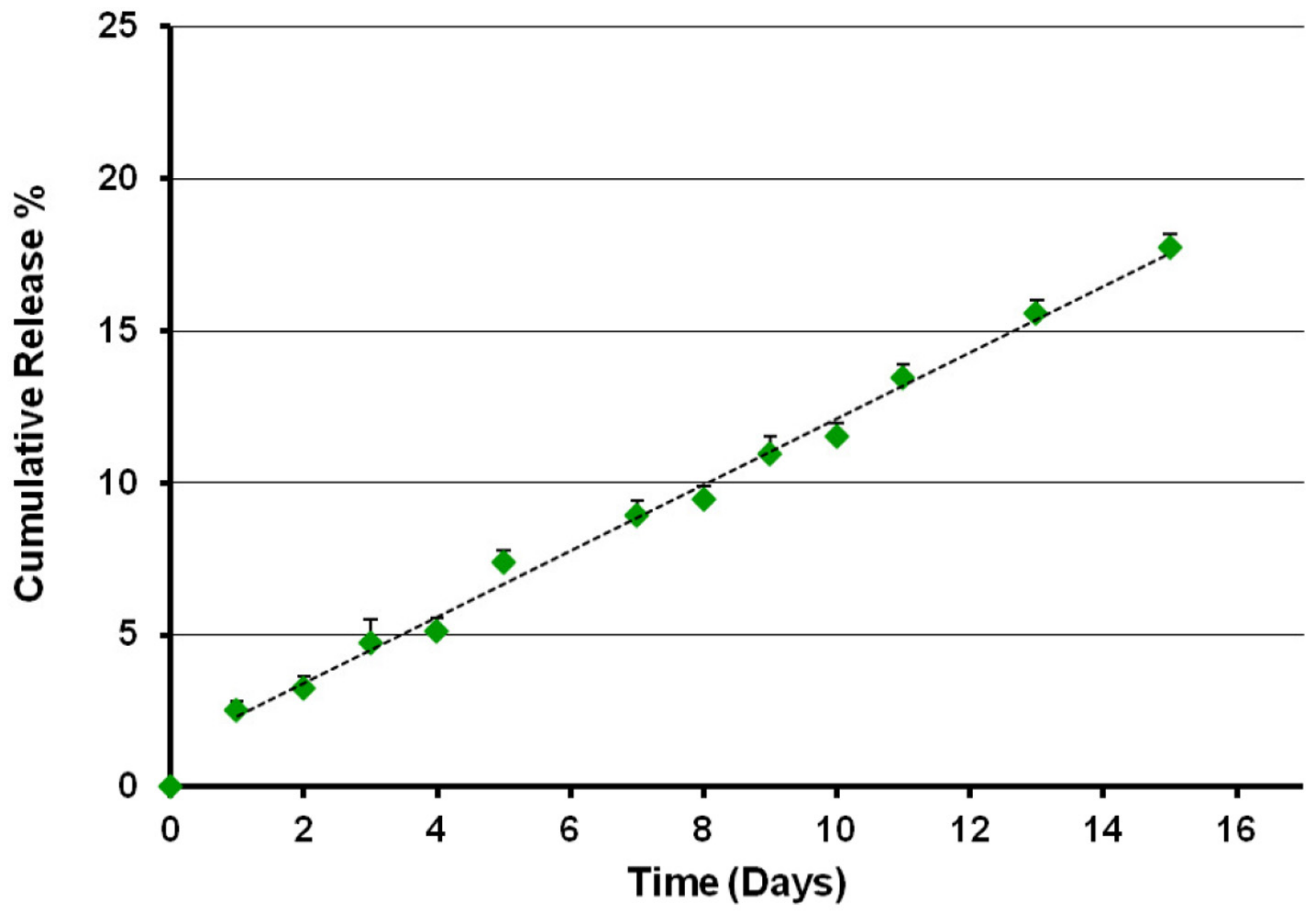
**Release profile of temporin B from TB-CS-NPs in SPB pH 7.4, 37°C**. After a short lag time, the system displayed a progressive linear release of the loaded active agent.

The TB loading capacity and encapsulation efficacy of CS-NPs, evaluated by means of Micro BCA^TM^ protein assay, are shown in **Table 2**. The results obtained revealed a TB loading of 4.8% and an encapsulation efficacy of 75%. The release kinetics of the peptide from TB loaded NPs was studied in SPB pH 7.4 for 15 days (**Figure 1**). After a first equilibration time (lag time), the system displayed a progressive linear release, according with the results obtained with the model peptide RSI.

### Cytocompatibility Evaluation of TB-CS-NPs vs. Free TB

**FIGURE 2:**
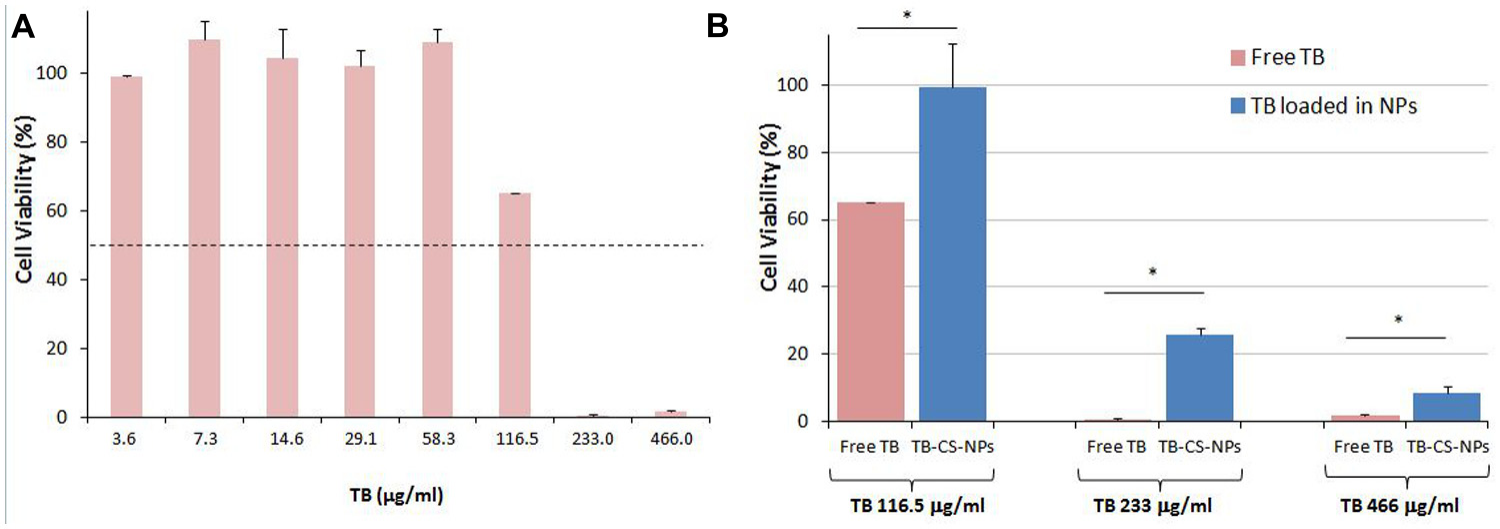
**Cytocompatibility evaluation of free TB **(A)** and of TB loaded into CS-NPs **(B)** in mouse embryo fibroblast cell line Balb 3T3/Clone A31. (A)** Determination of free TB cytotoxicity. Results show that free TB displays an IC_50_ value of ∼119 μg/ml after 24 h of incubation; **(B)** Comparison of cytotoxicity of different concentrations of free TB (116.5, 233, and 466 μg/ml) with the same amount loaded into CS-NPs (TB-CS-NPs), corresponding to TB-CS-NPs concentrations of 2.5, 5, and 10 mg/ml, respectively. Results show a statistically significant reduction of TB toxicity upon its loading into nanoparticles (^∗^*p* < 0.05, Student’s *t*-test).

The cytocompatibility of free TB and of the developed TB-CS-NPs was evaluated using mouse embryo fibroblast BALB/3T3 Clone A31 cell line as indicated in the International Standard ISO 10993- Part 5: “Test for cytotoxicity – *in vitro* methods.” In order to investigate the capability of the developed nanocarrier to limit the toxicity of the loaded TB against mammalian cells, at first determination of the IC_50_ of free TB was assessed. Results showed that TB generated an IC_50_ value of ∼119 μg/ml after 24 h of incubation (**Figure 2A**). Further investigations performed under the same experimental conditions employing TB loaded into CS-NPs highlighted a remarkable lower cytotoxicity of the bioactive compound (**Figure 2B**). In fact, by comparing the toxicity of different concentrations of free TB (116.5, 233, and 466 μg/ml) with the same amount loaded into CS-NPs, statistically significant differences on 3T3 cell viability were observed (*p* < 0.05, Student’s *t*-test).

### Bactericidal Activity of the Developed Nanocarrier Against *S. epidermidis*

Preliminary experiments were carried out in order to identify the optimal experimental conditions that could allow the assessment of TB antibacterial activity for prolonged intervals of times (data not shown). Addition of 1.25% TSB to the buffer assay was found to be the optimal concentration able to maintain the control bacteria alive for all the duration of the experiment, with no effect on TB antibacterial activity. In addition, a starting inoculum of approximately 1 × 10^7^ CFU/ml and a concentration of plain CS-NPs of 5 mg/ml were found to be optimal for monitoring the bactericidal activity for at least 4 days.

At these optimal conditions, the number of viable bacteria recovered after exposing the starting inoculum to TB-CS-NPs (5 mg/ml) for 4 h, 1, 2, 3, and 4 days, was compared to that obtained exposing the bacteria to plain CS-NPs (5 mg/ml) and to free TB. Taking into account the data collected from the *in vitro* release kinetics, performed under the same conditions of the bactericidal assays, the releasing rate of 5 mg/ml of TB-CS-NPs corresponded to 2.5 μg/ml/day. Therefore, the concentration of free TB (10 μg/ml) used in the bactericidal assay was approximately equal to the total amount of the peptide released by the loaded nanocarrier over the observational period (4 days). Bacteria incubated in the medium assay only served as control of cell-viability.

**FIGURE 3:**
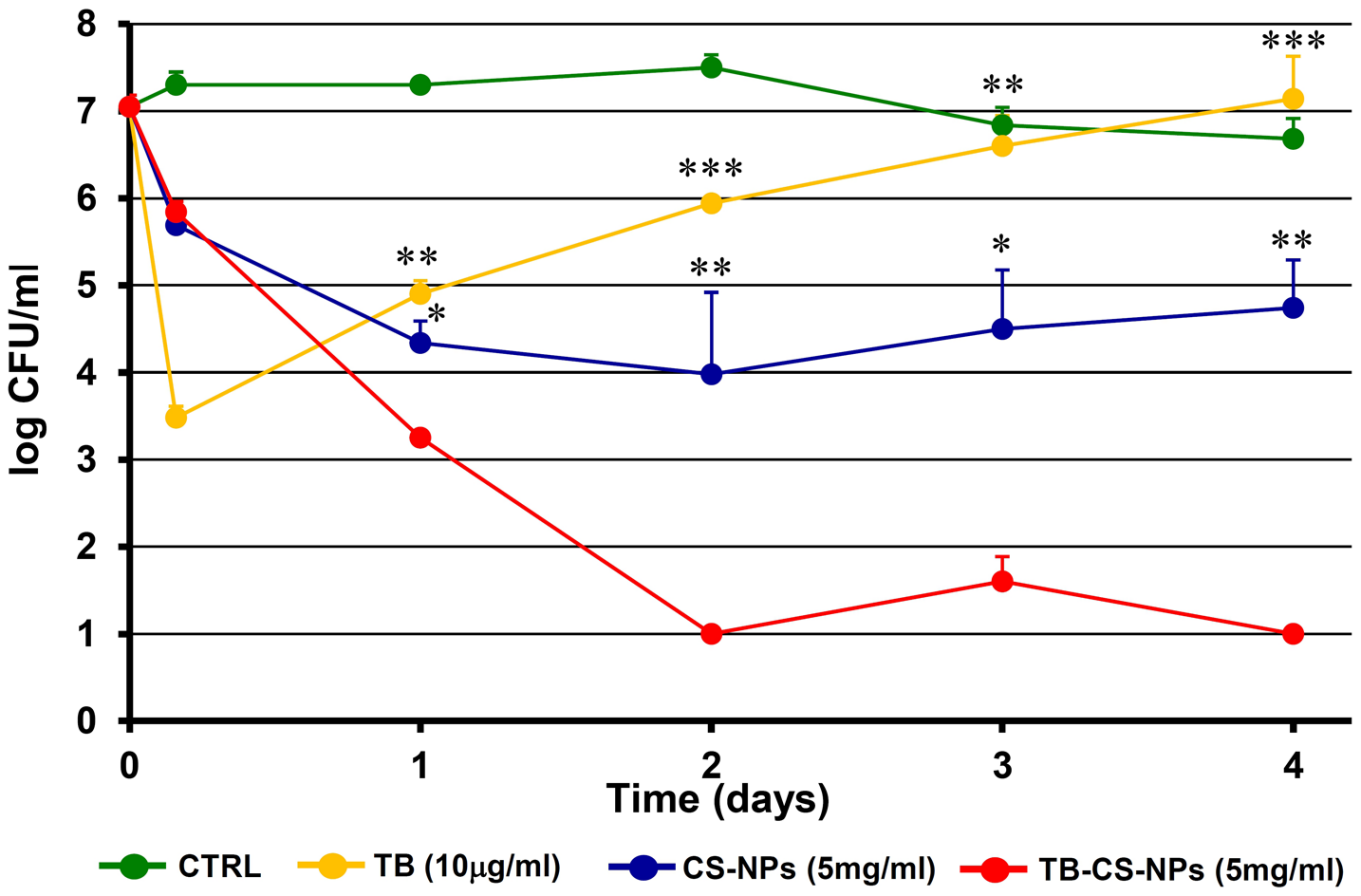
**Kinetics of the antibacterial activity of the developed nanoparticle-based AMP-delivery system against *Staphylococcus epidermidis* ATCC35984**. Bacterial cells were exposed for different time intervals to free TB, CS-NPs, or TB-CS-NPs. At the end of the exposure time aliquots of the cultures were serially diluted, plated on solid medium, and incubated at 37°C for 48 h before assessing the CFU count. Results are the mean values ± SEM of three independent experiments. CTRL, control bacteria incubated in the assay medium only. ^∗^*p* < 0.05; ^∗∗^*p* < 0.01; ^∗∗∗^*p* < 0.001 as compared to TB-CS-NPs, Student’s *t*-test.

**Figure 3** depicts the kinetics of the bactericidal effect against the reference strain *S. epidermidis* ATCC35984. At very early time points (4 h incubation) both CS-NPs and TB-CS-NPs exerted a modest bactericidal activity, while, as expected, free TB caused more than 3-log reduction in the number of CFU/ml as compared to not treated bacteria. Starting from 1 day of incubation, however, residual bacteria that have been exposed to free TB underwent to a progressive regrowth reaching, within 4 days of incubation, CFU levels comparable to those of control bacteria. In contrast, during the same incubation times, the TB-loaded nanocarrier (TB-CS-NPs) exerted a strong bactericidal activity against *S. epidermidis*, causing a statistical significant reduction in the number of CFU/ml, as compared to both free TB and plain CS-NPs. At 4 days of incubation, almost 4-log reduction in the number of viable *S. epidermidis* compared to plain CS-NPs, and 6-log reduction compared to free TB and control bacteria were observed.

**FIGURE 4:**
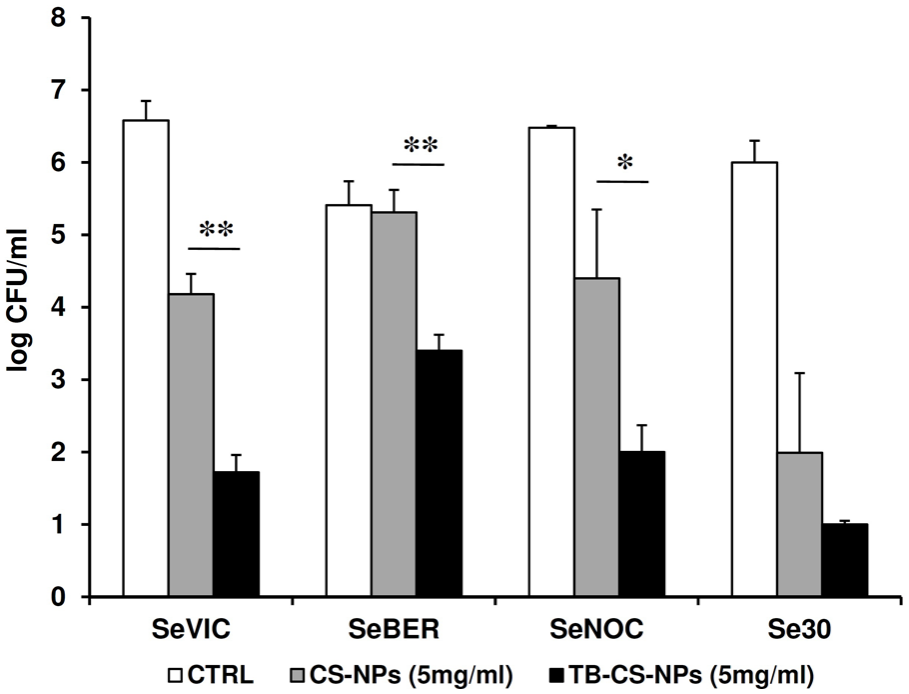
**Antibacterial activity of TB-CS-NPs as compared to the plain nanocarrier against clinical isolates of *S. epidermidis* after 4 days of incubation**. Bacterial cells were exposed for 4 days to plain CS-NPs or TB-CS-NPs. At the end of the exposure time aliquots of the cultures were serially diluted, plated on solid medium, and incubated at 37°C for 48 h before assessing the CFU count. Results are the mean values ± SEM of two independent experiments performed in duplicate. CTRL, control bacteria incubated in the assay medium only. ^∗^*p* < 0.05; ^∗∗^*p* < 0.01; as compared to TB-CS-NPs, Student’s *t*-test.

The antibacterial properties of the TB-CS-NPs as compared to plain CS-NPs was also tested against four clinical isolates of *S. epidermidis* after 4 days of exposure. As depicted in **Figure 4**, for three out of four strains, a statistically significant difference in the CFU number was obtained between plain and TB-loaded CS-NPs, similarly to what observed with the reference strain.

To test the hypothesis that the mere encapsulation of TB in CS-NPs could stimulate the antibacterial activity of chitosan, the peptide RSI was used as a control of the susceptibility assay as the peptide shares key chemical–physical properties with TB, but is not endowed with antibacterial activity. A preparation of RSI-loaded CS-NPs was obtained as previously described (Piras et al., 2015) and incubated at the concentration of 5 mg/ml for 4 days with *S. epidermidis* ATCC35984 cells. After the incubation period, the number of bacterial cells that survived the treatment was assessed and compared to that obtained incubating the same number of bacteria with plain CS-NPs, free RSI, or medium only. The number of CFU obtained following incubation with RSI-loaded CS-NPs was similar to that obtained with plain CS-NPs (4.1 ± 3 × 10^4^ versus 1.9 ± 0.6 × 10^4^ *p* > 0.05 Student’s *t*-test), suggesting that the entrapment of a cationic peptide do not enhance, *per se*, the antimicrobial properties of CS-NPs. As expected, no killing activity of free RSI was observed (data not shown).

## Discussion

Over the last few years, several nanoparticle-based antibiotic delivery systems have been developed and are under evaluation in clinical trials or have reached the market (Gao et al., 2011). Nevertheless, a real concern regarding the use of these nanosystems is that their therapeutic potential might be neutralized or highly reduced by the loss of efficacy of conventional antibiotics due to the growing rise of multi-drug-resistant microorganisms worldwide. Although bacterial mechanisms of resistance to AMPs have been described as well (Maisetta et al., 2011; Guilhelmelli et al., 2013), it is generally agreed that their development rate is much lower than that of conventional antibiotics, making AMPs promising future drugs against different types of infections (Easton et al., 2009; Batoni et al., 2011).

As the therapeutic efficacy and safety of AMPs administered by conventional methods is limited, their encapsulation in different kinds of delivery systems, aimed at improving their therapeutical potential, is being at the center of considerable attention. To date, AMPs have been mainly incorporated within micelles, liposomes, lipidic disks, and metal-based nanocomposites (Urbán et al., 2012; Carmona-Ribeiro and Dias de Melo Carrasco, 2014). However, over the last years, also numerous biocompatible and biodegradable polymers, both natural (e.g., alginate, CS, collagen, gelatin, hyaluronic acid) and synthetic (e.g., polyesters, polycarbonates, polyurethanes) have attracted increasing attention as matrices for the formulation of the AMPs carriers (Sobczak et al., 2013). Among all polymers, CS appears certainly one of the most promising to be used in the development of nanostructured antimicrobial systems because of its inherent antimicrobial properties and its ability to gel spontaneously on contact with multivalent polyanions under mild conditions. This feature allows the encapsulation of labile therapeutic macromolecules, such as AMPs.

In order to investigate the therapeutic potential of nanoparticle-based AMP delivery systems, in the present work TB was encapsulated into CS-NPs and the developed nano-system was finely characterized in terms of physical–chemical properties, releasing kinetics, toxicity, and antibacterial efficacy.

The TB-loaded CS-NPs were easily prepared by following the formulation parameters previously set for the CS-RSI model NPs (Piras et al., 2015). As expected, the obtained particles had positive surface charge, nanoscaled diameter and high peptide loading. Being an amphiphilic cationic peptide, TB is taking part to the ionotropic gelation in presence of the anionic TPP and the charge repulsion between cationic CS and cationic TB is reduced by the selected solution pH. The presence and participation of TB to the ionotropic gelation may induce conformational and charge rearrangements of CS that are reflected in a statistically significant reduction (*p* < 0.05, Student’s *t*-test) of the Zeta potential value. In agreement with previous studies (Piras et al., 2015), TB is progressively released by the system and the slope of the linear section of the release kinetics is comparable to that observed with the model RSI peptide.

Few examples can be found in the literature of AMPs successfully encapsulated in micro/NPs. Recently Chereddy et al. (2014) prepared PLGA NPs encapsulated with LL-37, with a mean diameter of 304 nm, by W/O/W emulsion–solvent evaporation technique. The LL-37 is the only human peptide belonging to the cathelicidin family, has powerful antibacterial activity, and is an amphipathic cationic helical peptide, features shared with TB, despite its longer chain (37 aa versus 13 aa of TB). The PLGA NPs had a low payload of peptide, 1.02 μg LL37/mg PLGA NPs, corresponding to 0.1% by weight. The evaluation of the release kinetics highlighted an initial burst release of LL-37 from PLGA-LL37 NP, corresponding to nearly 40% of total amount of loaded peptide within the first day, and reaching 80% of released peptide by day 14. The antimicrobial activity of the nanosystem was investigated *in vitro* against *Escherichia coli* only at early times of incubation (6 h), displaying a lower antimicrobial activity of the PLGA LL-37 loaded NPs compared to the free peptide.

In another report, LL-37 was loaded into CS/poly(g-glutamic acid) MPs with an average diameter of 1.2 mm. The MPs had a high loading % of LL-37 (40% by weight) as generally expected from MPs. Furthermore, the peptide was very quickly released, showing a burst value of 71.2% within the first hour and reaching a plateau of about 90% of cumulative release after 24 h. Despite the composite MPs were thought as a multifunctional carrier for LL-37 and nitric oxide to fight bacterial infections, the antimicrobial activity of the developed system was not assessed by the authors (Sun et al., 2015).

In the present work, the materials and formulation methods adopted for the loading of TB into CS based NPs allowed the achievement of a nanosized carrier with noteworthy peptide payload and release kinetics, with no burst effect and slow constant (linear) release rate.

The *in vitro* cytocompatibility evaluation of TB-CS-NPs highlighted the excellent role of the developed nanosystem in reducing TB toxicity profile toward mammalian cells. This result corroborates that the undertaken strategy of AMPs encapsulation in suitable nanocarriers may improve the potential of these therapeutic molecules by reducing their toxicity and enhancing their therapeutic activity by sustained and targeted delivery.

There are inherent experimental difficulties in the assessment of antibacterial activity for prolonged intervals of times mainly due to the fact that, after long incubation times bacterial cells undergo to spontaneous death because of nutrient limitation. An accurate evaluation of the different parameters involved was, therefore, necessary to identify the optimal conditions for the assessment of the antibacterial activity for prolonged intervals of times. In particular, the parameters studied included: (i) the identification of the minimal percentage of growing medium (TSB), to be added to the buffer assay (SPB, pH 7.4), able to provide sufficient amount of nutrients to ensure the vitality of control bacteria for all the observational period. In this regard, it is noteworthy to point out that antimicrobial activity of AMPs might be inhibited by the presence of the growth medium in the bactericidal assay that, therefore, has to be kept to the minimum; (ii) the entity of the starting inoculum able to ensure a detectable number of residual cells after the eventual initial burst; (iii) the optimal concentration of the empty CS-NPs; such concentration had to be high enough to allow the encapsulation of sufficient amount of TB, but not too high to mask the bactericidal activity of the released peptide when loaded NPs were used.

Given an inoculum of 10^7^ bacteria, we found that empty CS-NPs, used at a concentration of 5 mg/ml, were able to reduce the inoculum of approximately 2-logs as compared to the control bacteria incubated in medium only, thus contributing to the overall antibacterial activity of the nanosystem; in addition, based on the releasing kinetic studies, 5 mg/ml of TB loaded-CS-NPs were able to ensure a cumulative release of the peptide of about 10 micrograms in 4 days, corresponding to the TB bactericidal concentration.

For these reasons, we chose the concentration of 5 mg/ml as, for the chosen inoculum, it represented the optimal combination of CS and TB likely to allow both components of the nanosystem to exert their antimicrobial activity.

Interestingly, at the optimal experimental conditions identified, the TB-loaded nanocarrier was able to exhibit a strong antibacterial activity that lasted for all the observational period (4 days), and was statistically higher than that exerted by the plain CS-NPs or free TB. This long-lasting antibacterial activity was demonstrated not only against the *S. epidermidis* reference strains, but also against clinical isolates of *S. epidermidis* highlighting the potential of our delivery system as nanotherapeutic to treat bacterial infections in clinical settings.

The bactericidal action of CS has been widely investigated in recent years and it has been mainly attributed to its polycationic nature and consequent ability to interact with the anionic components of bacterial surface (Kong et al., 2010). This interaction would ultimately lead to membrane permeability, release of cellular contents and death.

The bactericidal effect of CS is highly dependent on several factors including its molecular weight and deacetylation degree, physical state, ionic strength, pH, surface characteristics of target microorganisms (Dash et al., 2011). Depending on one or more of these variables, alternative bactericidal mechanisms of chitosan have been proposed including inhibition of mRNA synthesis and DNA transcription (Sudarshan et al., 1992), chelating capacity (Rabea et al., 2003), induction of multiple changes in the expression profiles of genes involved in the regulation of stress, autolysis, and energy metabolism (Raafat et al., 2008).

Of note, CS-NPs were found to exhibit even a higher antimicrobial activity than the free polymer against both Gram-positive and Gram-bacteria; in this regard it has been proposed that thanks to their high surface charge density CS-NPs may be tightly adsorbed onto the bacterial surface and interact with bacteria to a greater degree than free chitosan (Qi et al., 2004).

Regarding the mechanism of action of TB, it seems to be dependent on its interaction with the microbial membranes, similarly to what has been demonstrated for many other AMPs (Mahalka and Kinnunen, 2009; Mangoni and Shai, 2009). The first step in this interaction is the electrostatic attraction between the cationic peptide and the negatively charged components of the bacterial cell wall. After that, the peptide reaches the inner membrane perturbing its permeability and causing a rapid cell death. The sharp drop in CFU number observed in our study, following incubation of bacteria with free TB is consistent with the above mentioned mechanisms of action of the peptide (i.e., bacterial membrane permeabilization). Inactivation of the free peptide and/or its sequestration by dead bacteria or medium components may explain the re-growth of the bacteria that survived the treatment observed at later times of incubation.

At the moment, the possible mechanism of the combined antibacterial activities of the TB-loaded CS-NPs developed in our study is not known. Our hypothesis is that, besides being bactericidal themselves, CS-NPs may deliver the encapsulated peptide directly to the bacterial surface meanwhile preventing its inactivation by interaction with medium components or dead bacteria; release of TB at the bacterial surface would allow the achievement of a high local concentration of the peptide that could rapidly reach the bacterial membrane causing cell death. If this was the case, even peptide concentrations below those needed to ensure a bactericidal effect of the free peptide would be sufficient to cause cell death.

A crucial issue regarding the release of antimicrobial compounds by delivery systems is the releasing kinetics. It is generally agreed that too fast releasing kinetics may provide a relatively high initial dose of the active drug, but also potential cytotoxicity and short-term action. In contrast, too slow releasing kinetics may not ensure the required therapeutic level of the antimicrobial compound, increasing the risk of selecting resistant cells that adapt to the environment. Therefore, an “ideal” antibacterial delivery system should exhibit a sufficiently high initial release rate (burst release) to counter any initial elevated infection risk, followed by a long period of drug release, within the therapeutically efficacious dosing zone, to eradicate bacteria that have survived the initial burst (Hetrick and Schoenfisch, 2006; Wu and Grainger, 2006). The results obtained from the assessment of the antibacterial activity of the nano-system developed in our study seem to perfectly match these requirements. In fact, the antibacterial activity exerted by the nanocarrier itself (CS-NPs) was able to ensure a “burst” activity within the first 24 h of incubation able to markedly reduce the starting inoculum. Afterward, TB was released in a linear manner by the nanocarrier and/or directly delivered to the bacterial surface was able to further reduce the viable count, preventing the regrowth of the residual cells and ensuring a long-lasting antibacterial activity.

Antimicrobial peptide delivery through nanocarriers, as the one developed in this study, has the potential to be employed in a wide range of applications. Pathological conditions that, in our opinion, could particularly benefit from the use of such nanosystems are those that occur at mucosal/skin surfaces and require multiple (daily) and long term drug administrations for prevention/treatment of microbial infections. These conditions include pulmonary infections in cystic fibrosis patients or wound infections in burned or diabetic patients. Besides being extremely time-consuming for the patients, the therapeutic regimen usually adopted in these conditions may enhance the risk of resistant mutant selection, contributing to the worldwide spread of bacterial resistance. The muco-penetrating ability of chitosan, the low tendency in inducing bacterial resistance of the delivered AMP, the high cytocompatibility and the strong antibacterial properties of both components, together with the continuous releasing kinetics are all properties that could likely contribute to the long-lasting control of microbial infections in the above mentioned conditions, meanwhile reducing the number of drug administrations and increasing the patient’s compliance.

In our study *S. epidermidis* was used as model Gram-positive bacterium. TB has previously shown activity also against a broad range of nosocomial Gram-positive and, to a lesser extent, Gram-negative species/strains (Mangoni et al., 2008). TB-analogs with improved activity against Gram-negative species have been recently described (Avitabile et al., 2013; Bezzerri et al., 2014). Thus, the nanocarrier described herein may represent a versatile tool to allow a long-term control of microbial infections sustained by different clinically relevant bacterial species.

## Conclusion

Research on the development of AMPs as new drugs may largely benefit from the use of nanotechnologies, hopefully allowing to overcome the limits that still hamper AMPs’ clinical use.

In the present work, we carried out a first characterization *in vitro* of a nanoparticle-based delivery system for AMPs with the aim to demonstrate the potential of such system in ensuring prolonged and strong bactericidal activity against a model bacterial species. In particular, we demonstrated that loading of TB, chosen as a model cationic alpha-helical AMP, in CS-NPs represents a promising and innovative strategy to ensure a long-lasting bactericidal activity against the clinically relevant species *S. epidermidis*. The results obtained also demonstrated that the nanocarrier itself contributed to the overall antibacterial activity of the developed nanosystem, allowing the achievement of an optimal killing kinetics, and reducing, at the same time, the toxic potential of the loaded peptide. *In vivo*/*ex vivo* studies will be needed to fully evaluate the ability of the developed nanosystem in preventing/treating infections occurring at mucosal/skin surfaces and in increasing the translational potential of promising peptide molecules.

## Author Contributions

AP, FC, and GB drafted the work; GM and LG acquired and analyzed the data regarding the antimicrobial susceptibility tests; AP, LG, and SS acquired and analyzed the data regarding the preparation and characterization of nanoparticles; MG and CB acquired and analyzed the data regarding cytotoxicity; AP, GB, GM, FC, and SE interpreted the data of the work. All the authors took part in revising the manuscript critically for important intellectual content, and approved the final version to be published. All the authors agree to be accountable for all aspects of the work ensuring that questions related to the accuracy or integrity of any part of the work are appropriately investigated and resolved.

## Conflict of Interest Statement

The authors declare that the research was conducted in the absence of any commercial or financial relationships that could be construed as a potential conflict of interest.

## Abbreviations

AMPs: antimicrobial peptides
ATTC: American Type Culture Collection
CFU: Colony forming units
CS: chitosan
CS-NPs: chitosan nanoparticles
DMEM: Dulbecco’s Modified Eagle Medium
MPs: microparticles
NPs: nanoparticles
PLGA: poly (lactic-co-glycolic acid)
RSI: Renine Substrate I
SPB: Sodium phosphate buffer
TB: temporin B
TB-CS-NPs: chitosan nanoparticles loaded with TB
TPP: sodium tripolyphosphate
TSA: tryptone soy agar
TSB: tryptone soy broth
